# Epidemiology of myasthenia gravis in the United States

**DOI:** 10.3389/fneur.2024.1339167

**Published:** 2024-02-16

**Authors:** Yun Ye, Dana Jorgensen Murdock, Chao Chen, Wolfgang Liedtke, Caitlin A. Knox

**Affiliations:** ^1^The Division of Epidemiology, The Ohio State University, Columbus, OH, United States; ^2^Regeneron Pharmaceuticals, Inc., Tarrytown, NY, United States

**Keywords:** epidemiology, incidence, myasthenia gravis, prevalence, retrospective

## Abstract

**Introduction:**

Global studies of epidemiology of myasthenia gravis (MG) have pointed to increasing prevalence of this rare autoimmune disorder affecting the neuromuscular synapse; however, no new data for the USA were available for decades. We aimed to estimate the incidence rate and prevalence of MG in a large-scale insured US population.

**Methods:**

We conducted a population-based retrospective cohort study to estimate the annual incidence and prevalence of MG cases in the USA during 2017. Using a previously validated algorithm, we identified cases of MG in two Truven Health MarketScan databases, which during 2017 included a sample of approximately 20 million commercially insured and Medicare recipients, plus 10 million Medicaid recipients. We report crude incidence and prevalence and calculated age-and sex-standardized estimates for the USA based on the 2017 American Community Survey. We estimated the number of adult cases during 2021 by extrapolating from the stratified estimates to the population size from the 2021 American Community Survey.

**Results:**

From the US commercially/Medicare-insured cohort, we calculated an age-and sex-standardized incidence of 68.5 new cases per million person-years with an adjusted prevalence of 316.4 per million. Within the Medicaid-insured population, similar yet slightly lower numbers emerged: the adjusted incidence was 49.7 new cases per million person-years, and the adjusted prevalence rate was 203.7 cases per million. Given our results, we were able to estimate that there were approximately 82,715 US adults living with MG in 2021 (or an estimated 320.2 cases per million adults in the USA). We observed a strong effect of age and sex when stratifying the identified incidence rate and prevalence, with a pattern of female preponderance among the younger age brackets, a male preponderance for older cases in the commercially/Medicare-insured cohort, and the disease incidence and prevalence steadily increasing with age.

**Discussion:**

Our updated US population-based estimates of MG epidemiology demonstrate an increase in the previously reported incidence and prevalence from over 20 years ago, in keeping with developments in westernized, industrialized countries. Notable findings of steadily increasing prevalence with age, driven by robust increases in elderly males, prompts questions for basic-translational research, therapeutics, and public health.

## 1 Introduction

Myasthenia gravis (MG) is a rare autoimmune synaptopathy of the neuromuscular junction, which causes peripheral motor weakness with fatiguability. Incidence and prevalence of MG have been studied globally for several decades, yielding considerable variations across studies (incidence range: 1.7–28 cases per million person-years; prevalence range: 15–329 per million) (1–7). Over time prevalence estimates have been increasing, in keeping with increased prevalence of other autoimmune disorders, which is perhaps reflective of increased liability to autoimmunity via external factors that impact the immune system of genetically predisposed individuals, for example through epigenetic mechanisms facilitated by pollution, xenobiotics, climate change, nutritional changes, changes of microbiome, and many more (8). But the changing epidemiology of MG might also be due to improved awareness of the disease, earlier diagnosis, and extended patient life expectancy (1–4, 9, 10). While there are several global estimates of MG, epidemiologic data have been derived from larger geographically representative datasets. Furthermore, the prevalence and incidence of MG in the USA have not been examined for over two decades, and have never been investigated in a large geographically representative claims-based dataset.

As previously noted, only a limited number of studies have been conducted in the USA. While informative, these prior US studies had inherent limitations which may lead to bias when extrapolating to the current US population. For example, they either focus on select populations (i.e., small geographic areas) (11, 12) or examine MG crisis only (13), thus are selected for a particular phase/status of the disorder and may not be generalizable to the entire US population. Additionally, there is a need to further elucidate racial differences which have been previously suggested (12). To date only one study, in 2013, has provided population-based incidence and prevalence estimates of MG for North America (3). This study applied a previously validated algorithm within the Canadian healthcare system (Ontario Health Insurance Plan), which covers 95% of the population, to identify newly diagnosed patients with MG in the province of Ontario, Canada. The estimated incidence and prevalence of MG for 2013 among the approximately 11.3 million people in the Ontario healthcare system was 23 per million person-years and 263 per million, respectively.

Given the reported increase in the prevalence and incidence of MG at other locations globally, there is a need to provide a contemporary estimate of MG in the USA to support public health, guide basic and translational research, and ultimately facilitate the provision of better adapted medical care to patients living with MG. Therefore, the objective of this study was to assess the epidemiology of MG in the USA using large representative populations, and to explore age-, sex-, and race-specific differences in incidence and prevalence.

## 2 Methods

### 2.1 Study design and data source

Administrative claims data have been leveraged to describe the epidemiology and natural history for multiple diseases, including several rare diseases (3, 14, 15). For rare diseases that have specific International Classification of Disease (ICD) codes, such as MG, administrative claims data are a useful source that can identify cases hard to characterize in other sources, capture early or mild cases, and include patients diagnosed and treated in primary healthcare settings, nursing homes, and other long-term care facilities by utilizing inpatient and outpatient diagnoses, lab charges, prescriptions, and basic demographics.

To calculate the 2017 annual incidence and annual prevalence of MG, we utilized data from January 1, 2016, to December 31, 2018, from three large US administrative claims databases: Truven Health MarketScan US Commercial Claims and Encounters Database; the Medicare Supplemental and Coordination of Benefits Database; and the Multi-State Medicaid Database. The first two databases covered healthcare service use within fee-for-service plans among an employed population and their families, as well as Medicare-eligible employees and their families with employer-sponsored supplemental plans, together representing approximately 20 million individuals in 2017. In the commercial and Medicare databases, information on US census regions (Midwest, Northeast, South, and West) were available. Race and ethnicity were not reported in either of these databases. Due to similar data structures and patient demographics, these two databases were combined for the analysis.

In the USA, Medicaid provides healthcare coverage to millions of Americans, including eligible low-income individuals of all ages as well as people with disabilities. Medicaid is administered by states, according to US federal requirements, and eligibility can vary state by state. The MarketScan Medicaid Database included over 10 million enrollees in 2017, with Medicaid coverage under fee-for-service and managed care plans from numerous geographically dispersed states. While information on geographic location for enrollees is not available, most had information on race/ethnicity for the following categories: non-Hispanic White, African American, Hispanic, and other race.

The databases were de-identified and housed on the Instant Health Data analytic platform (version 589a602a, Panalgo, Boston, MA), where the analyses were performed. No identifiable protected health information as specified by the Health Insurance Portability and Accountability Act of 1996 was extracted, and the study protocol was exempt from institutional review board review at the Ohio State University (Study ID: 2020E0966).

### 2.2 Identification of patients with MG

To identify patients with MG, we utilized a previously validated algorithm from Breiner et al. (16) which was developed using the Ontario administrative healthcare data. The optimal algorithm from Breiner identified a patient with MG if the patient had ≥ one inpatient diagnosis, or ≥ five outpatient diagnoses and a test code for single-fiber electromyography or Tensilon test within 1 year, or ≥ three dispensations of pyridostigmine within 1 year (16). This algorithm performed well within the administrative healthcare data sources of this Canadian study, with sensitivity = 81.6%, specificity = 100%, positive predictive value = 80.0%, and negative predictive value = 100%. Furthermore, it provided an accurate prevalence estimate within the validation cohort with the highest correlation statistics (*κ* = 0.81) among all the algorithms proposed (16). Due to the potential off-label use of pyridostigmine for orthostatic hypotension, systemic sclerosis, and other conditions (albeit uncommon), we required a diagnosis of MG at any time during the study period for those who qualified for the algorithm based on prescription of pyridostigmine, to improve the positive predictive value of the algorithm (17–20). Continuous enrollment was required from 1 year before (baseline) the first event of interest (diagnosis, test code, or dispensation of pyridostigmine) in 2017 until the last event in the algorithm for an individual to be identified as an incident patient with MG.

For our analysis, all patients with MG were described demographically by age on January 1, 2017, and by sex, region (for commercial or Medicare databases only), and race (Medicaid database only); the Charlson Comorbidity Index score was calculated based on the presence of specific ICD codes in the claims during the 2017 calendar year (21).

Because the algorithm we utilized was validated in a Canadian administrative healthcare database, we conducted several sensitivity analyses to explore the impact of modifying the algorithm to identify patients with MG. The analyses included requiring only one hospitalization; one hospitalization or two out-patient visits; and one hospitalization or two outpatient visits and a prescription claim. Additionally, we conducted a sensitivity analysis using the best performing algorithm from a recently validated US algorithm examining prevalence among patients aged ≥65 years, with Medicare insurance, and receiving care from any Cleveland Clinic Ohio facility. This algorithm used two office visits separated by at least 4 weeks, or one hospital admission and one office visit separated by 4 weeks (sensitivity = 80.0%, specificity = 99.98%, positive predictive value = 80.0%, and negative predictive value = 99.98%) (22).

### 2.3 Statistical analyses

Characteristics of incident and prevalent patients with MG were described and compared. *p*-values were reported from unequal variance two-sample *T*-tests for continuous variables, and Chi-squared tests for categorical variables.

The annual incidence and annual population-based prevalence were calculated among those aged ≥18 years on January 1, 2017. Incident patients with MG were identified in 2017 if they had no evidence of MG (in-patient, out-patient, testing, or prescription claims associated with MG) during 2016. The person-time at risk was defined as the time accrued from January 1, 2017, to identification of MG case, disenrollment, or the end of 2017, whichever came first. For period-prevalence estimation, all patients with MG were identified in 2017, regardless of whether they had any events in 2016. Individuals who were enrolled for at least 1 day in 2017 were included in the total population for the prevalence calculation toward the denominator.

Age-and sex-specific rates were computed for each 10-year age category and standardized to the US adult population according to the 2017 American Community Survey (23). Age- and sex- standardized rates were calculated as the sum of expected cases in the standard population in each category (the crude rates per category multiplied by the standard population), divided by the total standard population. For each age category, sex-adjusted rates were calculated as [(crude rates for males multiplied by the percentage of males in the standard population) plus (crude rates for females multiplied by the percentage of females in the standard population)] (24). The spatial pattern was also examined by mapping the annual prevalence of patients with MG in 2017 by census region for commercial and Medicare data only (25). Regional prevalence was compared to the one with the lowest prevalence using a Chi-squared test. Subgroups with <five individuals were suppressed in reporting.

To estimate the total number of adult cases in the USA during 2021, we used the age-, sex-, and insurance (i.e., commercial/Medicare, Medicaid, and uninsured/others)-stratified crude estimates and multiplied each crude prevalence estimate by the Annual Social and Economic Supplement of the Current Population Survey population estimates for 2021. To avoid double counting of dual Medicaid/Medicare enrollees, the number of patients who were 65+ years old was derived from commercial and Medicare Advantage data only. Prevalence for those who were < 65 years old and were uninsured/others were estimated by averaging the commercially insured and Medicaid population. We were then able to extrapolate this estimate to calculate a single prevalence estimate for MG in the USA for 2021.

## 3 Results

As noted in our methods section, we identified patients with MG if the patient had ≥1 inpatient diagnosis, or ≥ 5 outpatient diagnoses and a test code for single-fiber electromyography or Tensilon test within 1 year, or ≥ 3 dispensations of pyridostigmine within 1 year. From the US commercially/Medicare-insured cohort, we calculated that the age-and sex-standardized incidence of MG was 68.5 new cases per million person-years, with an adjusted prevalence of 316.4 per million.

**Figure 1:**
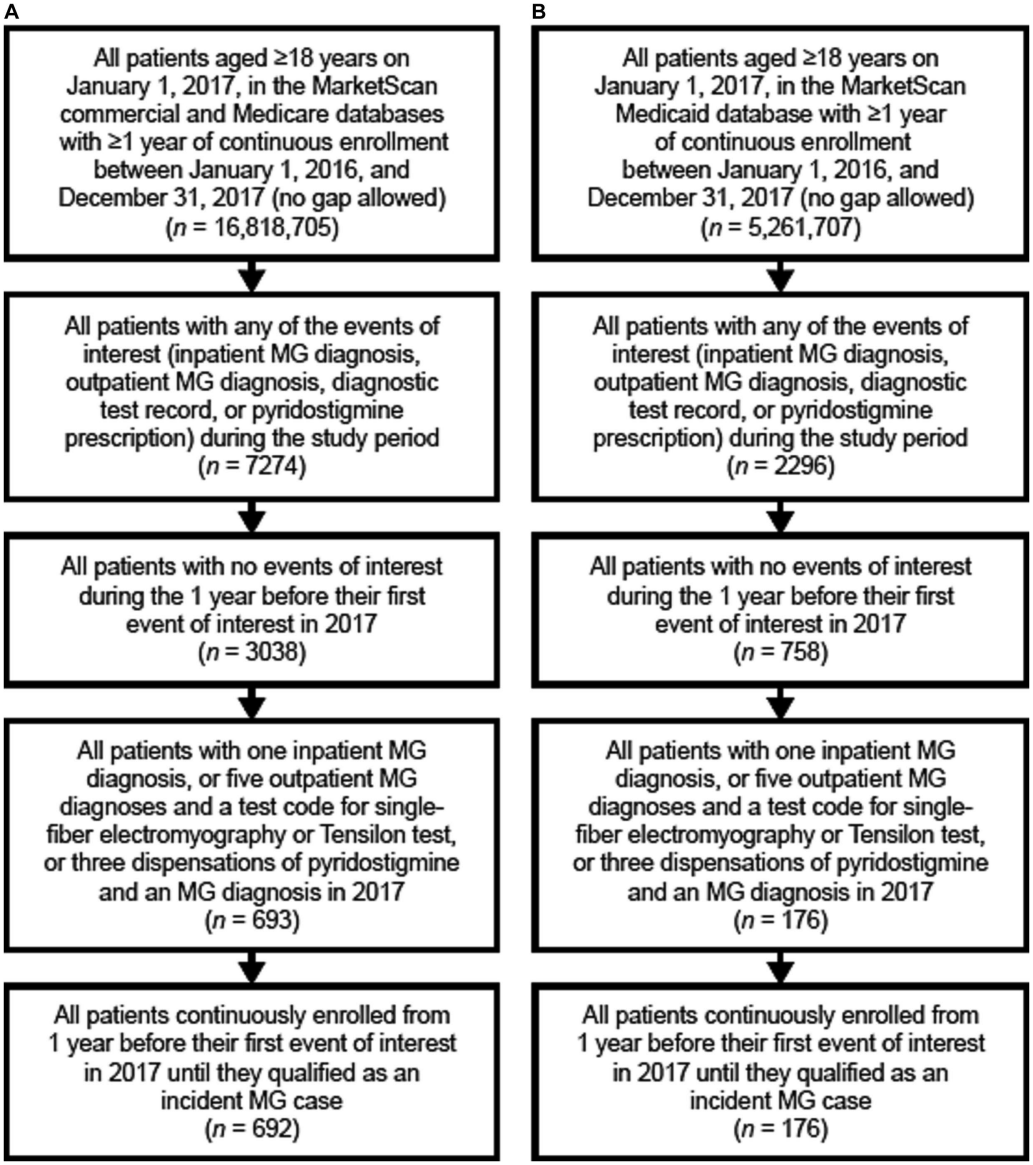
Flow chart of the identification of incident MG cases in panel **(A)** the commercial and Medicare claims databases, and **(B)** the Medicaid database. MG, myasthenia gravis.

When exploring the descriptive characteristics of the Medicaid database as compared to the commercial/Medicare database, there was a higher proportion of incident cases of MG in the Medicaid database for patients who were aged >65 years, female, and had more comorbidities (Figure 1; Table 1).

**Table 1:** Characteristics of incident and prevalent MG patients in 2017.

	Commercial and Medicare claims databases		Medicaid claims database	
	Incident (*n* = 692)	Prevalent (*n* = 4,187)	Incident (*n* = 176)	Prevalent (*n* = 1,266)
Age, years, mean (SD)	59.47 (16.1)	59.08 (16.5)	59.97 (17.8)	54.56 (19.2)
Age group, years, *n* (%)^a,b^				
18–34	48 (6.9)	356 (8.5)	16 (9.1)	237 (18.7)
35–44	71 (10.3)	436 (10.4)	17 (9.7)	178 (14.1)
45–54	119 (17.2)	707 (16.9)	26 (14.8)	201 (15.9)
55–64	236 (34.1)	1,293 (30.9)	44 (25.0)	263 (20.8)
≥65	218 (31.5)	1,395 (33.3)	73 (41.5)	387 (30.6)
Sex, *n* (%)^a,c^				
Female	361 (52.2)	2,262 (54.0)	123 (69.9)	878 (69.4)
Male	331 (47.8)	1925 (46.0)	53 (30.1)	388 (30.6)
US region, *n* (%)^a^				
Midwest	139 (20.1)	875 (20.9)		
Northeast	80 (11.6)	526 (12.6)		
South	309 (44.7)	1,640 (39.2)		
West	56 (8.1)	383 (9.1)		
Unknown	108 (15.6)	763 (18.2)		
Race, *n* (%)				
Non-Hispanic White			97 (55.1)	654 (51.7)
African American			62 (35.2)	458 (36.2)
Hispanic			– (−)	26 (2.1)
Other			15 (8.5)	110 (8.7)
Charlson Comorbidity Index score, mean (SD)^d^	1.76 (2.14)	1.43 (2.02)	2.83 (2.49)	1.96 (2.17)

^a^*p* > 0.05, χ^2^ test comparing incident vs. prevalent MG cases in the commercial and Medicare claims databases.

^b^*p* < 0.001, χ^2^ test comparing incident vs. prevalent MG cases in the Medicaid claims database.

^c^*p* > 0.05, χ^2^ test comparing incident vs. prevalent MG cases in Medicaid claims database.

^d^*p* < 0.001, *t*-test comparing incident vs. prevalent MG cases.

MG, myasthenia gravis; SD, standard deviation.

### 3.1 Incidence rates

**Figure 2:**
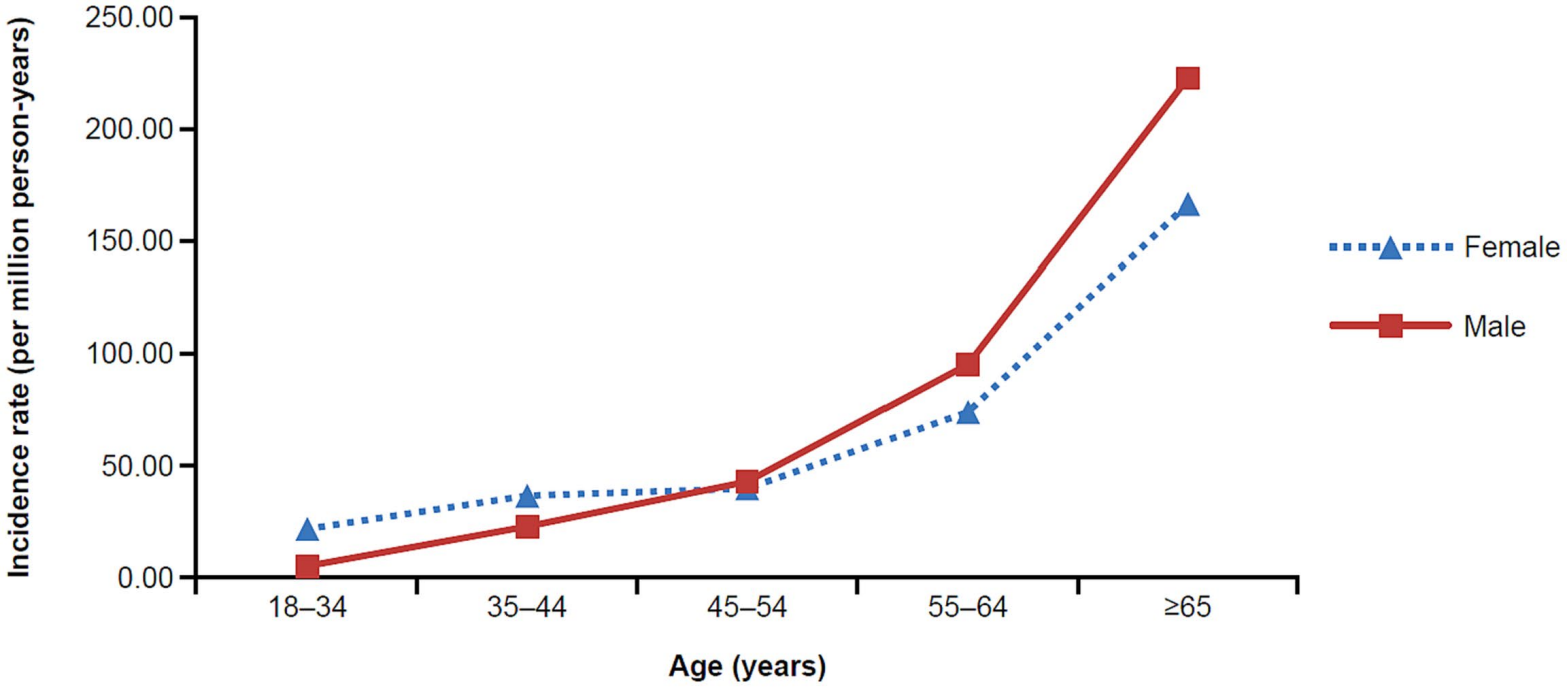
Incidence rate of MG in the commercial and Medicare claims databases by age and sex (2017). MG, myasthenia gravis.

The unadjusted MG incidence rate was 54.55 and 47.67 per million person-years, respectively, for the Commercial/Medicare and Medicaid databases (Tables 2, 3). However, when stratifying by sex, age, and race (Medicaid only), differences were observed. Specifically, there was a higher incidence in females as compared to males in both databases until the age of 54 (Tables 2, 3). Surprisingly, in the commercial/Medicare database we observed a shift, as males had robustly higher incidences after the age of 55, powerfully rising with age (Figure 2). When adjusted for age and sex, the incidence rate was calculated to be 68.48 and 49.68 per million person-years, respectively.

**Table 2:** Incidence rate and prevalence estimates of MG in the USA from commercial and Medicare claims data (2017).

	2017 US population (ACS)		Incidence (cases per 1,000,000 person-years)				Prevalence (cases per 1,000,000 persons)			
	Females	Males	Females	Males	Overall	Standardized^a^	Females	Males	Overall	Standardized^a^
All	129,341,135	122,729,360	53.89	55.30	54.55	68.48	202.19	191.37	197.07	316.35
Age group, years										
18–34	37,214,265	38,571,882	21.79	5.24	13.69	13.37	75.03	22.35	49.48	48.22
35–44	20,646,170	20,471,735	36.66	22.83	30.22	29.78	143.66	67.97	108.06	105.98
45–54	21,440,066	20,890,889	39.86	42.92	41.30	41.38	197.26	116.93	159.46	157.62
55–64	21,745,371	20,274,405	74.00	95.29	83.94	84.28	280.14	335.07	305.81	306.65
≥65	28,295,263	22,520,449	166.54	222.83	191.89	191.49	782.27	1,333.88	1,030.27	1,026.74

^a^Standardized to the 2017 ACS US population.

ACS, American Community Survey; MG, myasthenia gravis.

**Table 3:** Incidence rate and prevalence estimates of MG in the USA from Medicaid claims data (2017).

	2017 US population (ACS)		Incidence (cases per 1,000,000 person-years)				Prevalence (cases per 1,000,000 persons)			
	Females	Males	Females	Males	Overall	Standardized^a^	Females	Males	Overall	Standardized^a^
All	129,341,135	122,729,360	51.67	40.40	47.67	49.68	194.75	158.13	181.85	203.67
Age group, years										
18–34	37,214,265	38,571,882	12.88	11.80	12.52	12.33	91.72	58.97	81.42	75.05
35–44	20,646,170	20,471,735	30.62	24.37	28.47	27.51	182.87	105.39	155.84	144.30
45–54	21,440,066	20,890,889	61.65	21.42	45.29	41.80	244.20	160.61	209.02	202.95
55–64	21,745,371	20,274,405	68.73	77.36	72.40	72.89	292.54	261.06	278.61	277.35
≥65	28,295,263	22,520,449	123.74	94.28	114.89	110.68	392.06	372.04	385.73	383.19
Race										
Non-Hispanic White	81,892,645	78,232,690	50.08	51.67	50.64	50.86	197.96	156.76	183.51	177.83
African American	16,514,154	14,546,359	58.67	31.40	49.63	45.90	221.04	163.32	202.31	194.01
Hispanic	20,055,692	20,240,231	18.19	34.45	23.81		115.86	185.83	137.82	
Other			43.56	20.88	33.78		130.69	147.52	137.84	

^a^Standardized to the 2017 ACS US population. Standardized rate only reported for non-Hispanic White and African American people.

ACS, American Community Survey; MG, myasthenia gravis.

Exclusively in the Medicaid database, we were able to explore the relationship between incidence of MG by race. Overall, the incidence ranged from 23.81 to 50.67 per million person-years and was highest among non-Hispanic Whites and lowest among Hispanics. We extrapolated to the age-, sex-, and insurance-stratified estimates to calculate that there were approximately 17,417 new cases of MG among US adults in 2021 (*n* = 324,356,000).

### 3.2 Prevalence of MG

**Figure 3:**
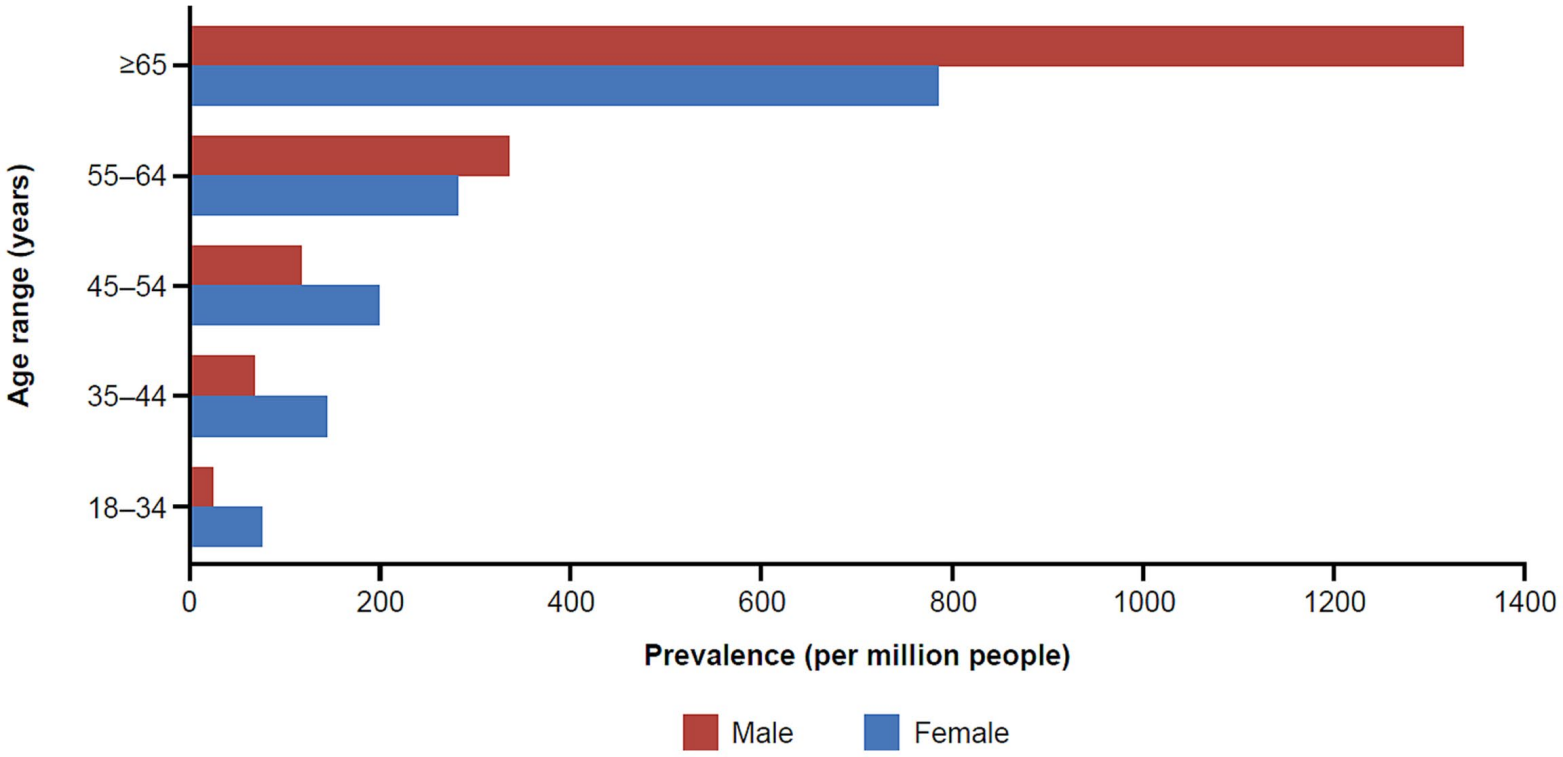
Prevalence of MG in the commercial and Medicare claims databases by age and sex (2017). MG, myasthenia gravis.

Similar to results for the incidence rates, we observed a difference in prevalence by age and sex (Figure 3). One striking finding in Medicare/Insured was a vastly increased prevalence in older males aged ≥65 years of 1333.88, the overall highest prevalence in the entire study. We calculated a standardized prevalence by age and sex of 316.35 and 203.67 per million people in the commercial/Medicare and Medicaid databases, respectively (Tables 2, 3).

**Figure 4:**
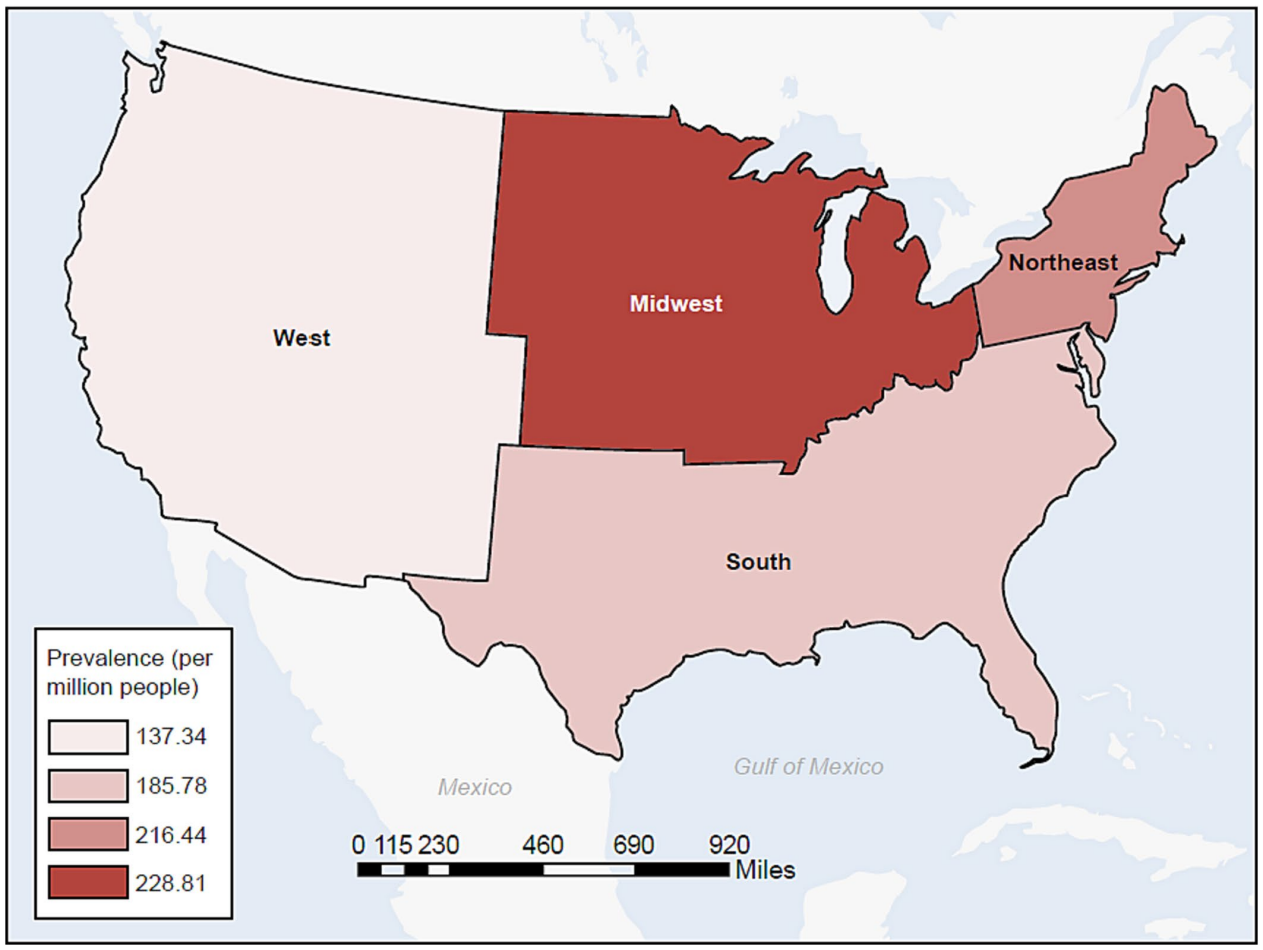
Prevalence of MG in the commercial and Medicare databases by census region (2017). MG, myasthenia gravis.

In the commercial/Medicare database, we further explored the prevalence of cases of MG by region (Figure 4). In a peculiar geographic pattern, the prevalence of MG ranged from 137.34 to 228.81 per million population, and was lowest in the West and highest in the Midwest. Again, in Medicaid, we were able to explore the relationship of prevalence and race/ethnicity; we found that African Americans had the highest prevalence among the racial/ethnic groups studied and Hispanics had the lowest, at 202.31 and 137.82 per million, respectively (Table 3). When further examining race/ethnicity by sex, African American females had the highest incidence rate and prevalence among all groups at 58.7 per million person years and 221.04 per million, respectively.

Finally, extrapolating the age-, sex-, and insurance-stratified estimates to the 2021 US population, we calculate that there were approximately 82,715 US adults living with MG in 2021, leading to an overall estimated prevalence of 320.2 cases per million people. Additionally, we predict that 66.2% of the patients in 2021 would be aged ≥65 years and 52.8% would be male.

### 3.3 Sensitivity analyses

In order to test the robustness of the algorithm, we conducted several sensitivity analyses to evaluate the potential impact of using different algorithms by varying the required codes for case identification. With the original case identification, using the Canadian algorithm (16), we identified a crude incidence of 54.55 per million person-years and a crude prevalence of 197.07 per million persons. The sensitivity analyses found a crude incidence range of 26.57–47.62 per million person-years and a crude prevalence range of 70.70–387.83 per million persons. We observed that only requiring one hospitalization led to much lower estimated crude incidence and prevalence rates of 26.57 and 70.70, respectively. However, only requiring two outpatient visits, without a required time between visits, may overestimate the actual number of MG cases. This is supported by the algorithms conducted by Breiner et al. which reported a positive predictive value of 9.2 (6.2–11.8). We do note that other algorithms from Lee et al. reported a better positive predictive value for two outpatient visits (79.2); however, they restricted the observed period to 2 years and a single health system which limits the variability of coding and generalizability of the results. We were restricted to a 1-year period due to limitations of our data source, thus the positive predictive value is not known and supports our rationale for utilizing the optimal algorithm identified by Breiner et al. (16).

## 4 Discussion

To date, our study used the largest population-based data source to estimate the incidence and prevalence of MG in the USA. The age- and sex- standardized incidence rate among US adults was 68.48 per million person-years among a commercially- or Medicare-insured population, and 49.68 per million person-years in a Medicaid-insured population. The adjusted prevalence estimates were 316.35 and 203.67 cases per million people in the two databases, respectively, thus in a range of findings as previously reported (2, 3). We extrapolated this to calculate the overall US prevalence of 320.2 per million people and an incidence of 54 per million person-years. We observed a steady increase of both metrics with age, and observed the well-known female preponderance for MG at <55 years of age, then an unexpected robust further increase driven by males who are taking over females for >55 years of age. This pattern of older males having higher MG prevalence than all other subgroups has not been reported previously for any autoimmune disease, and is also unprecedented when compared with previous MG epidemiology findings. In comparison with other MG epidemiology studies conducted globally, we position the USA at the upper end of MG prevalence, in keeping with an overall trend of increasing incidence/prevalence of MG and autoimmunity in general.

In addition, in our study, the commercially or Medicare-insured population had higher incidence and prevalence rates compared to those who were Medicaid-insured. Since we utilized insurance databases, MG cases were identified based on their healthcare utilization. Medicaid covers low-income and disabled Americans aged <65 years (26). Medicaid coverage has been reported to be associated with more emergency department visits and fewer outpatient visits than private marketplace coverage among low-income adults, even after adjusting for socio-economic status (27). Lower utilization of ongoing medical care services may partially explain the lower estimates of incidence and prevalence rates among the Medicaid-insured population.

**Figure 5:**
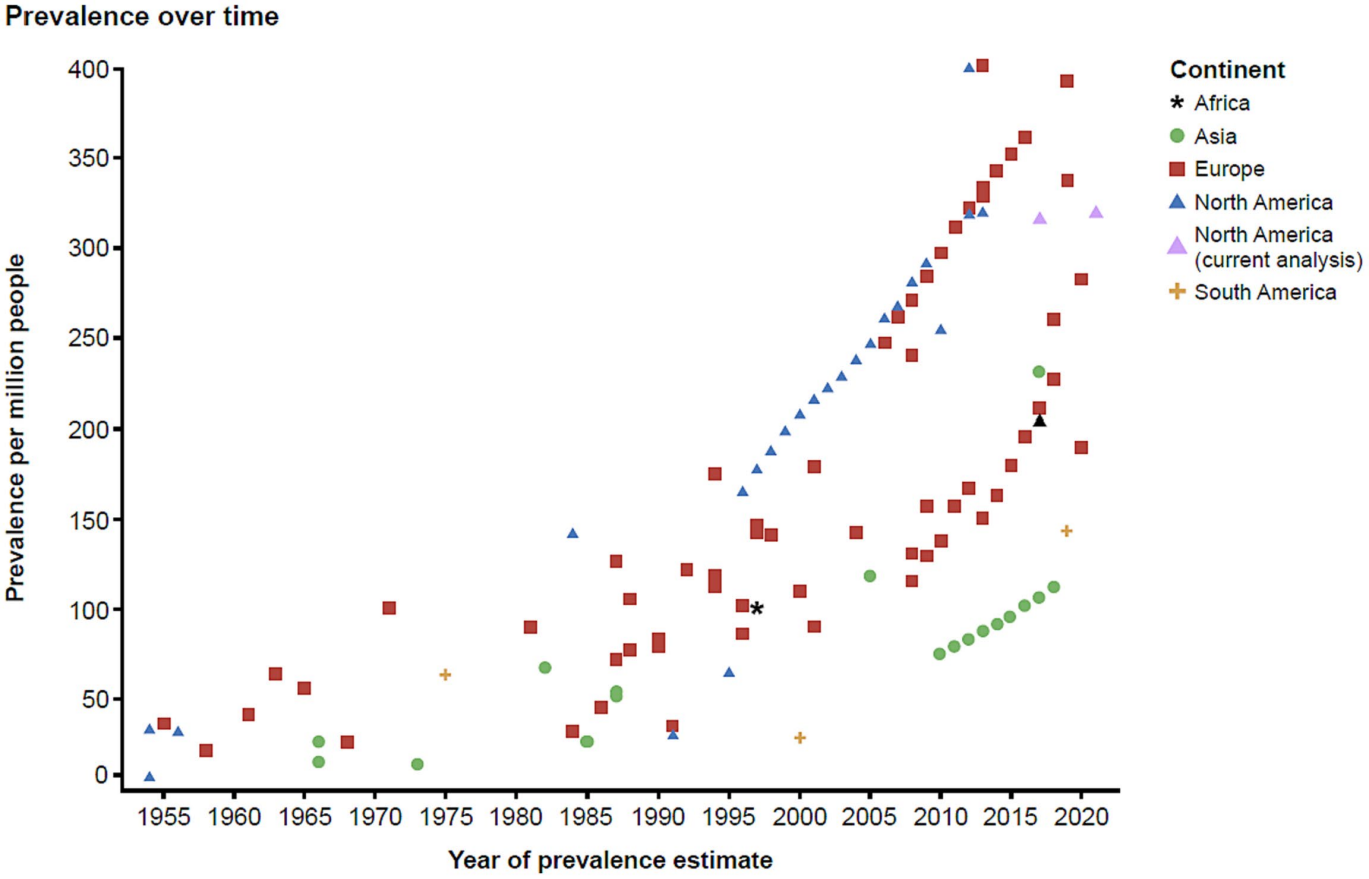
Global prevalence of MG over time. MG, myasthenia gravis.

To contextualize the findings of this study with the current evidence in the literature, we conducted a targeted literature review. The findings from the literature are summarized in a scatterplot (Figure 5) which was stratified by continent, also indicating the estimated approximate US prevalence for 2021. These data illustrate that since 1955 there has been an increase in the reported prevalence of MG globally.

Our estimates are even higher than those previously reported, and the underlying causes contributing to this increasing trend need to be further evaluated (3–6, 13). The typical female preponderance in age brackets under 55 years points toward female gonadal steroids facilitating autoimmunity, as in many other autoimmune diseases. However, the observed increase in prevalence in males aged >55 years was very surprising, and potential explanations may include factors favoring an increase in aging males, e.g., vascular risk factors, obesity, and lifestyle factors such as physical inactivity (28). Physical inactivity means less active neurotransmission at the neuromuscular synapse of the non-active muscles, and thus it can negatively affect neuromuscular synapse functions. These inactive neuromuscular synapses could thus become a more likely target for immune surveillance if there is a propensity for autoreactive immune surveillance. A relative decrease in prevalence in females at the same time could possibly be attributed to lowered female hormonal impact in menopause. Interestingly, MG epidemiology findings with similar trends in older males and females were also noted very recently in a smaller regional cohort in Germany (29). However, we did not observe the female-to-male shift starting with age 55 years in the Medicaid population, where females consistently had increased prevalence regardless of age. This could be due to the source population of the database, as females are more likely to be insured in Medicaid.

Our study provides novel data on the racial differences in the epidemiology of MG in the USA, leveraging the Medicaid database. Only two previous MG epidemiology studies have previously examined different racial and ethnic groups (12, 13). Due to the availability of racial/ethnic data, we were only able to assess racial differences within the Medicaid database, which may not be representative of the US population. Our current results show that patients who identified as African American had higher standardized prevalence than those who identified as non-Hispanic White, but a lower standardized incidence rate. Alshekhlee et al. (13) reported a similar pattern, in that African American females had the highest incidence rate using data on inpatient visits from 2000 to 2005.

Studies which utilize administrative data have limitations, and our current study is no exception. We understand that misdiagnosis or misuse of coding in administrative claims may affect the validity of our estimates. However, the validated algorithm we used incorporated a mandate for diagnostic redundancy from different settings, test records, and dispensation of medications (pyridostigmine), which improved our confidence identifying MG cases and minimized the impact of misclassification due to misdiagnosis or misuse of coding. Second, we understand that the algorithm that was utilized was restrictive, as our priority was interval validity, and therefore may not be generalizable to the broader MG population and may not appropriately capture the US standard of care. Thus, we conducted a sensitivity analysis using a recently validated algorithm for the case identification of MG that showed a similar pattern of crude incidence and prevalence as compared to our original estimates. Third, the Truven database that was utilized in this study only captures information when people are actively insured. Segmented insurance episodes may have affected our ability to identify patients with MG, specifically because multiple events were required to qualify a case (except a diagnosis based on inpatient care). A sensitivity analysis was conducted among those with continuous insurance coverage since 2013 in the commercial and Medicare database that showed similar pattern of incidence rate as compared to our original estimates.

Bearing in mind the limitations stated above, we believe our study provides a much-needed epidemiological update of the incidence rate and prevalence of MG in the USA. To date, our study is the first to explore the incidence and prevalence in the USA within databases that cover large geographically and socioeconomically diverse populations. Our study provides evidence of an increase in the incidence and prevalence of MG in the USA over time. Of note, we observed higher crude rates in older males compared to females in MG prevalence in the commercial/Medicare population, epidemiology findings that require additional mechanistic explanations from laboratory-guided science. Such enhanced understanding will form the basis of rationally based MG clinical management strategies which will be needed given the apparently increasing societal burden of the disease, with projected further increased impact in an aging society, perhaps pointing toward personalized medicine approaches tailored to specific sub-cohorts.

## Data availability statement

The original contributions presented in the study are included in the article, further inquiries can be directed to the corresponding author.

## Ethics statement

Ethical review and approval was not required for the study on human participants in accordance with the local legislation and institutional requirements. Written informed consent from the patients/participants or patients/participants' legal guardian/next of kin was not required to participate in this study in accordance with the national legislation and the institutional requirements.

## Author contributions

YY: Conceptualization, Formal analysis, Methodology, Writing – review & editing. DM: Conceptualization, Formal analysis, Methodology, Writing – review & editing. CC: Conceptualization, Formal analysis, Methodology, Writing – review & editing. WL: Conceptualization, Formal analysis, Methodology, Writing – review & editing. CK: Conceptualization, Formal analysis, Methodology, Writing – review & editing.
